# Using Adjuvants to Drive T Cell Responses for Next-Generation Infectious Disease Vaccines

**DOI:** 10.3390/vaccines9080820

**Published:** 2021-07-24

**Authors:** Rekha R. Rapaka, Alan S. Cross, Monica A. McArthur

**Affiliations:** Center for Vaccine Development and Global Health, University of Maryland School of Medicine, 685 West Baltimore Street, Baltimore, MD 21201, USA; across@som.umaryland.edu (A.S.C.); mmcarthu@som.umaryland.edu (M.A.M.)

**Keywords:** T cell, adjuvant, differentiation, antigen presenting cell, CD8+ T cell, CD4+ T cell, Th, memory, TLR, APC

## Abstract

Using adjuvants to drive features of T cell responses to vaccine antigens is an important technological challenge in the design of new and improved vaccines against infections. Properties such as T helper cell function, T cell memory, and CD8+ T cell cytotoxicity may play critical roles in optimal and long-lived immunity through vaccination. Directly manipulating specific immune activation or antigen delivery pathways with adjuvants may selectively augment desired T cell responses in vaccination and may improve the effectiveness and durability of vaccine responses in humans. In this review we outline recently studied adjuvants in their potential for antigen presenting cell and T cell programming during vaccination, with an emphasis on what has been observed in studies in humans as available.

## 1. Introduction

Adjuvants are substances that enhance the immune response to a vaccine. Adjuvants may occur naturally as part of a vaccine, as seen in the pathogen associated molecular patterns (PAMPs) contained in live-attenuated vaccines or may specifically be added to a vaccine preparation to enhance immune responses. Additionally, certain antigen delivery platforms may have adjuvant functions. For example, antigen delivery platforms, such as adenoviral vectors have intrinsic PAMPs, and liposomes may be modified to impact antigen processing pathways, enhancing the immune response to the vaccine antigen. Currently, aluminum salts, followed by oil-in-water emulsions, are the most common adjuvants in clinical use [1,2]. Adjuvants are hypothesized to exert their effects through stimulation of the innate immune system, with innate immune cell activation and cytokine and chemokine production impacting cellular recruitment and activation at the site of vaccine administration. Adjuvants may stimulate the innate immune system through cellular pattern recognition receptors (PRR) and activation of signal transduction pathways in antigen presenting cells (APCs), such as tissue resident dendritic cells. APC programming by different adjuvants may impact features of APC maturation and migratory function towards draining lymph nodes and other secondary lymphoid organs. In the process of presentation of antigen by APCs, APCs impact T and B cell priming within lymph nodes, key processes in vaccine-elicited adaptive immune responses. Adjuvants may also impact the longevity of antigen in the body, assisting antigen in forming a depot and enhancing antigen presentation through an extended period of immune cell stimulation.

Next-generation vaccines involve precisely defined antigens, often involving short mRNA or DNA sequences, specific polysaccharide conformations, or modified proteins. Advances in technologies for developing vaccine antigens, including methods to stabilize protein conformations as used in the SARS-CoV-2 mRNA vaccines [3], or focusing antigen epitopes to enhance T cell responses or skew the T cell repertoire [4], are in development. Despite advances in antigens used in vaccines, including novel platforms, and the use of structural biology and systems biology to design highly specific responses to antigens [5], the optimization and utilization of adjuvants has proceeded more slowly. Improvements in our understandings of adjuvants may impact aspects of T cell differentiation upon vaccination, and potentially the development of long-term immunologic memory.

T cells hold a critical role in shaping adaptive antibody responses in the setting of vaccination and infection. T cells regulate properties of evolving immune responses, such as the development of immunologic memory, and have direct effector functions against infected cells. The functions of T cells in vaccine-elicited responses are important and likely go far beyond optimizing affinity matured class-switched antibody responses in existing vaccines. Advancements in adjuvant science may allow selective activation of specific innate immune sensors in triggering specific inflammatory pathways in APCs, driving selected pathways of immune responses that may be more optimal for specific vaccines. Greater understanding of how T cell responses can be shaped by adjuvants may significantly improve the efficacy and longevity of protection in next-generation vaccines.

## 2. Induction of Cytotoxic CD8+ T Cell Responses by Adjuvants

As demonstrated in a variety of different infection models, and particularly for pathogens with a prominent intracellular lifecycle, CD8+ T cells may play a central role in host defense against infection [6,7,8,9,10,11]. Differentiation of antigen-specific effector and memory CD8+ T cells could be utilized in vaccine-mediated immune protection, impacting the detection and clearance of infected cells. CD8+ T cells directly kill cells that are identified as infected or abnormal through recognition of major histocompatibility class I (MHC I) complexed to peptide at the host cell surface. During an infection, APCs play a critical role in priming antigen naïve CD8+ T cells, through the presentation of antigen, and through cellular and receptor-mediated co-stimulation or inhibition, leading to the proliferation and differentiation of antigen specific CD8+ T cells with specific migratory and cytotoxic functions [12]. Enhancement of intracellular antigen presentation with strategies that emphasize intracellular antigen phases, such as with use of saponins, with DNA or RNA based vaccination, or with viral vector-based antigen presentation, may improve CD8+ T cell responses [13,14,15].

Saponins, derived from the bark of tree *Quillaja saponaria* or from the leaves of *Quillaja brasiliensis*, are a family of amphipathic adjuvants with surfactant properties. The *Quillaja saponaria* saponins when isolated by size and weight were found to have fractions which were immunogenic and not associated with toxicity in murine models, such as QS-7 and QS-21 [16,17]. Given the favorable features and relative abundance of QS-21, many studies of saponins as adjuvants has focused on this particular fraction. Saponin QS-21, when formulated in cholesterol containing liposomes, has been shown to enhance human monocyte dendritic cell uptake of antigen through a receptor independent, cholesterol dependent mechanism [18]. Additionally, QS-21, was found to accumulate in and destabilize lysosomes, increasing in a dose-dependent manner the production of cellular activation inflammatory cytokines IL-6 and TNF-α [18]. It is hypothesized that this process of antigen presentation, with antigen escape from the lysosome and entry into the cytosolic space, and antigen presentation of peptides on MHC I by dendritic cells, enhances priming of cytotoxic T cells in lymphoid tissue.

The value of saponins in eliciting cytotoxic CD8+ T cells in humans and animal models is illustrated from studies in the field of anti-cancer vaccines. For example, the NY-ESO-1 protein antigen emulsified in the ISCOMATRIX adjuvant (described in [19], composed of purified fractions of saponin, phospholipid and cholesterol resulting in particles with a 40 nm cage-like structure) demonstrated an ability to mature human monocyte derived dendritic cells, and these dendritic cells demonstrated a capacity to cross present HLA-A2 restricted epitopes of NY-ESO-1 to CD8+ T cells [20]. In humans with melanoma, when ISCOMATRIX adjuvanted NY-ESO-1 protein was given as a vaccine, vaccinees developed high frequencies of long lasting specific CD8+T cell responses in vivo on booster immunization to a wide array of NY-ESO-1 peptides [21]. Cytotoxic CD8+T cell responses have been observed in murine models of malignancy with protein vaccines adjuvanted with ISCOMATRIX [22], including lysis of human cancer cell lines expressing tumor antigen [20]. Extrapolating from studies in the field of cancer, infections with a major intracellular component during the life cycle, as in viral infections, or infections with intracellular bacteria such as *Mycobacterium tuberculosis* or parasites, such as *Plasmodium falciparum*, may particularly have benefit from saponin-based adjuvants in vaccine design.

Saponins as adjuvants in infectious disease models have shown promise in promoting CD8+ T cell differentiation. In a model of bovine viral diarrhea virus (BVDV) vaccination in mice, mice vaccinated with BVDV antigen together with *Q. brasilensis* saponin had significantly increased production of CD8+ T cells producing the effector cytokine IFN-γ [23]. In mice immunized with Ebola virus glycoprotein, the Matrix-M adjuvant, derived from mixing 2 specific ISCOMATRIX types (as described in [24]), was demonstrated to significantly enhance production of antigen specific CD8+ T cells responses, compared to Ebola virus glycoprotein vaccine adjuvanted with alum, or with no adjuvant [25]. In this model, multifunctional CD8+ T cells, here CD8+ T cells simultaneously producing two or three of the cytokines, TNF-α, IL-2, or IFN-γ, were observed at increased frequencies if Ebola glycoprotein antigen was administered with Matrix-M, and occurred in a Matrix-M dose-dependent manner. In a murine model of Ebola infection, where CD8+ T cells are required for protection from infection [26], and where there is general precedent for multifunctional T cells in viral infections as limiting disease progression [10,27,28,29], this qualitative aspect of increased multifunctional CD8+ T cells observed with saponin adjuvant may be important for vaccine efficacy. Similarly, in a murine model of vaccination with the NVX-CoV2373 for SARS-CoV-2, the SARS-CoV-2 spike protein adjuvanted with Matrix-M led to the induction of increased CD8+ T cells producing cytokines, as well as multifunctional CD8+ T cells when compared to immunization of the spike protein without adjuvant [30]. Additionally, baboons immunized with NVX-CoV2373, if adjuvanted with Matrix-M, had increased production of IFN-γ cells in peripheral blood mononuclear cells stimulated with NVX-CoV2373. In humans, Matrix-M adjuvant has recently been evaluated with the Ebola virus glycoprotein for safety and immunogenicity [31], and has also been utilized in large scale trials in humans of the SARS-CoV-2 spike glycoprotein NVX-CoV-2373 vaccine during the COVID-19 during the pandemic [32,33]; studies of CD8+ T cell responses in humans for these vaccines are ongoing.

Enhancing production of cytotoxic CD8+ T cells may also be imparted by the delivery vehicle carrying the antigen, such as with liposomes. Liposomes consist of lipid bilayers encasing an aqueous core and may vary in size, charge, and lipid composition among other features [34]. Liposomes are notable for their ability to protect antigens from degradation, interact with cell membranes and optimize antigen internalization, and to carry a wide variety of antigens with lipophilic or hydrophilic properties. The biochemical composition of liposomes may impact their ability to enhance T cell priming. Vaccination with liposomes containing unsaturated fatty acids coupled to protein antigen in mice led to increased induction of antigen specific CD8+ T cells, in addition to CD4+ T cells; while if the liposomes only contained saturated fatty acids, only antigen specific CD4+ T cells were induced and CD8+ T cells were not significantly induced [35]. It was further demonstrated in this study by confocal microscopy that liposomes containing unsaturated fatty acids effectively targeted processing pathways leading to the cytosol, outside of lysosomes, which would more readily allow for peptide presentation onto MHC class I [35]. Hence, the specific composition of liposomes may direct the process of APC processing of vaccine antigen, impacting the processes of antigen presentation directly, as well as the process of APC maturation. Sterically stabilized liposomes (SSL), or liposomes containing large molecules, such as polyethylene glycol, coupled with protein antigen, have been shown in human dendritic cells to induce proliferation of CD8+ T cells at a rate of 300 times the rate of proliferation of CD4+ T cells [36]. Increased CD8+ T cell generation is hypothesized to occur as more antigen delivered by these liposomes was observed to be present in the cytosol compared to within lysosomes, with cytosolic antigen processed by transporter associated with antigen-processing 1 (TAP1) dependent pathways. Dendritic cells (DC) from TAP1 knock-out mice stimulated with protein antigen and complexed to SSL had significantly impaired antigen-specific CD8+T cell proliferation compared to DC from wild-type mice [36]. These studies collectively demonstrate that liposomes may enhance CD8+ T cell induction by impacting APC processing of antigen, and that the specific molecular composition of liposomes may be manipulated to selectively drive CD8+ T cell responses.

The first mRNA vaccines in use in humans for prevention of COVID-19, BNT162b2 (Pfizer) and mRNA-1273 (NIAID-Moderna) make use of an antigen delivery strategy similar to that of liposomes, specifically with lipid nanoparticles (NP) containing cholesterol, polyethylene glycol to enhance stability, and cationic lipids which bind and stabilize mRNA [37]. Lipid NP may range in size from 10–1000 nM, are designed to be endocytosed, and have a solid particulate nature. Lipid NP are optimized to carry nucleic acids, and in contrast to liposomes, do not necessarily have an aqueous core. mRNA based vaccines have good precedent for generation of antigen specific CD8+ T cells [38,39,40]. Both BNT162b2 and mRNA-1273 vaccines elicit SARS-CoV-2 spike glycoprotein antigen specific CD8+ T cells in humans. The mRNA vaccine BNT162b2 elicits high levels of IFN-γ producing CD8+ T cells in the peripheral blood specific for the spike glycoprotein receptor binding domain, which were observed after booster immunization; these frequencies were also notably higher than as observed in individuals who had developed and recovered from COVID-19 [41]. Interestingly, in this study, a higher dose of the vaccine, but without booster dosing, led to very low levels of CD8+ T cells responses, suggesting the critical nature of booster vaccination to enhance antigen specific CD8+ T cell frequencies in the blood. With mRNA-1273, spike glycoprotein specific CD8+ T cells were also observed in volunteers after booster immunization [42]. In a separate study where BNT162b2 was administered to volunteers, followed by booster at 22 days, several volunteers developed multifunctional CD8+ T cells with persistence of antigen-specific memory CD8+ T cells in the blood through at least 85 days of monitoring [43].

The live attenuated yellow fever vaccine and smallpox vaccinia vaccine induce high frequencies of multifunctional CD8+T cells in humans, with increased antigen load and pattern recognition receptor activation hypothesized to contribute to the effect [44,45,46,47]. Viral vector-based vaccines, where a virus is used as an antigen expression platform, with additional gene alterations to ensure reduced virulence and/or replication incompetence also have precedent for induction of T cell responses including CD8+ T cells. Several new viral vector-based vaccines have come into clinical use in humans recently, including for Ebola hemorrhagic fever and for COVID-19 [48,49,50,51]. The role of CD8+T cells in host defense may be central in prevention of infection; for example, in a small cohort of cynomolgus macaques vaccinated with recombinant adenovirus 5 encoding Ebola virus glycoprotein, 4 of 5 macaques were not protected if CD8+ T cells were specifically depleted in vivo prior to Ebola challenge [52]. The Ad26.COV2.S and ChAdOx1 COVID-19 vaccines both elicit spike glycoprotein antigen specific CD8+ T cells in humans [51,53].

Adjuvants with the capacity to stimulate intracellular compartment-associated toll-like receptor 9 (TLR9) or toll-like receptor 3 (TLR3) may also have promise in enhancing development of CD8+ T cells upon vaccination in humans. CpG, which are unmethylated cytosine and guanine oligonucleotide motifs, are commonly found in viruses and bacteria. There are four types of synthetic CpG adjuvants, with different immunostimulatory properties [54]. Recognition of CpG occurs at the cellular level with activation of TLR9 within APCs. It is important to note that differences in CpG ligands and interaction with TLR9 diverge between mice and humans, hence, the relevance of CpG as an adjuvant for a specific vaccine must be gathered in primate models prior to approaching studies in humans. Macaques immunized with a plasmid DNA vaccine containing the simian immunodeficiency virus *tat* gene and also containing synthetic CpG sequences, developed high levels of Tat protein-specific CD8+ T cells [55]. Mechanistically, this effect was thought to be at least partially driven by the CpG sequences and their ability to stimulate the early innate immune response, as macaques vaccinated with the vector containing unmethylated CpG without *tat* insertion had significantly reduced simian/human immunodeficiency virus replication on challenge compared to macaques not receiving any vaccination. In patients with melanoma, serial vaccination with CpG oligonucleotides combined with melanoma antigen A protein along with incomplete Freund’s adjuvant led to the induction of tumor antigen specific CD8+ T cell responses [56]. However, frequencies of melanoma A specific CD8+ T cells were reduced by 1-fold if CpG was not included in the vaccine. Importantly, the specific CpG structure may have differential effects on human APCs and their interactions with CD8+ T cells, as observed in studies with human PBMCs and the impact of different CpG on naïve vs. memory CD8+ T cells [57].

Polyinosinic:polycytidylic acid (poly(I:C)) is a TLR3 agonist and a synthetic double-stranded RNA mimicking viral nucleic acid, that has also been investigated for potential adjuvant effect in enhancing CD8+ T cell responses during vaccination. In a model of murine Ebola infection, a filovirus-like particle vaccine adjuvanted with poly (I:C) increased the efficacy of the vaccine in reducing filovirus levels on challenge and led to increased multifunctional CD8+ T cell responses to the vaccine, compared to vaccination without poly (I:C) [58]. In Langerhans cells derived from patients with human papillomavirus 16 (HPV16) infection and advanced cervical intraepithelial neoplasm, there were significantly enhanced inflammatory responses on exposure to HPV16 virus-like particles (HPV16 cVLP) if co-cultured with stabilized poly(I:C) [59]. Further, Langerhans cells stimulated with HPV16 cVLP and stabilized poly(I:C) on co-culture with autologous CD8+ T cells had significantly increased production of IFN-γ producing antigen-specific CD8+ T cells compared to culture without stabilized poly(I:C) [59]. Poly(I:C) has also been demonstrated to further enhance the immunostimulatory potential of live-attenuated vaccines, which already contain several intrinsic PAMPs. For example, in a murine model of live attenuated influenza vaccination, treatment with poly(I:C) after vaccination led to increased dendritic cell activation and increased CD8+ T cell responses within the lung [60]. These observations underscore the potential of CpG and poly(I:C) as adjuvants in enhancing multifunctional, antigen-specific CD8+ T cell responses at systemic sites, and at the mucosa. More translational studies are needed to establish the benefit of poly (I:C) in promoting CD8+ T cell responses in humans.

Collectively, saponins, liposomes and lipid NP, viral vectors, and certain TLRs agonists may play critical roles in impacting antigen presenting cells to prime and differentiate antigen specific CD8+ T cells during vaccination (see Table 1). Future vaccines will further develop these and similar modalities in settings where CD8+ T cell dependent functions are critical in human defense against infection.

## 3. Induction of T Follicular Helper Cell Responses by Adjuvants

The disruption of T follicular helper (Tfh) cell responses is associated with an impaired antibody response to infection or vaccination in numerous models [61], and enhancement of Tfh production in vaccination correlates with improved B cell memory in response to vaccination [62]. Tfh cells are involved in germinal center formation and processes of B cell affinity maturation and selection. Tfh selection of B cell clones producing somatically hypermutated, high-affinity antibodies is a central process in B cell memory development. Tfh are defined by their localization in germinal centers, are associated with cell surface markers CXCR5, ICOS, PD-1, and CD40L, and produce high quantities of cytokines IL-4 and IL-21. While these cells are not easily surveyed in humans given their localization during an immune response within central lymphoid tissues rather than the peripheral blood, circulating memory Tfh-like cells have been characterized in a variety of infectious disease models [63,64]. In humans, these cells display a T central memory cell phenotype (CCR7+/CD45RA-), express lymph node B cell zone homing molecule CXCR5, and are able to stimulate B cell plasma cell differentiation and immunoglobulin production in vitro [64].

Increased induction of Tfh has been associated with certain adjuvants in development, in particular adjuvants with capacity to activate the toll-like receptor 4 (TLR4) innate immune cell signaling pathway. The glucopyranosyl lipid adjuvant-stable emulsion (GLA-SE) adjuvant, developed by the Infectious Diseases Research Institute in Seattle, has been associated with increased Tfh induction in human vaccination. Glucopyranosyl Lipid A (GLA) is a synthetic toll-like receptor TLR4 agonist which binds to the MD2 portion of TLR4 in humans, and as GLA-SE is delivered as an oil (squalene) in water stable nano-emulsion (SE). In a clinical trial of volunteers receiving an experimental malaria peptide vaccine, there were significantly increased frequencies of Tfh induced when the peptide vaccine was adjuvanted with GLA-SE compared to when adjuvanted with alum [65]. Further, volunteers receiving the vaccine with the GLA-SE adjuvant compared to alum had a longer-lived antibody response, as well as a heightened extrafollicular antibody response to the peptide antigen; and the use of GLA-SE was also associated with induction of specific Tfh clonotypes in humans [65]. Here, the direct choice of adjuvant impacts the properties of adaptive CD4+ T cells responses in humans, with dominant Tfh clonotypes elicited, as well as long-lived antibody responses. Similarly, in mice immunized with recombinant protein, the GLA-SE adjuvant enhances production of Tfh compared to alum, SE alone, or GLA without SE, and the GLA-SE adjuvant is associated with increased memory B cell development on vaccination [66].

A similar adjuvant, GLA-LSQ, which contains the GLA adjuvant but emulsified in a liposomal adjuvant containing QS21 saponin (LSQ), enhances Tfh responses in the setting of peptide immunization in mice [67]. Here, Tfh were found in increased frequencies in draining lymph nodes in mice immunized with a peptide with GLA-LSQ adjuvant compared to an aluminum-based adjuvant. Given increased localization of Tfh to lymph nodes during a vaccine-elicited immune response, this supports the concept that use of GLA-LSQ adjuvant during antigen presentation enhances Tfh presence at sites impacting the process of antibody affinity maturation. Booster immunization with peptide antigen combined with GLA-LSQ adjuvant demonstrated even further increase in Tfh in draining lymph nodes, occurring in association with significant quantities of antigen specific plasmablasts, which were notably absent in the mice receiving the aluminum-based adjuvant [67].

TLR4 agonist monophosphoryl lipid A (MPLA), derived from bacterial lipopolysaccharide and similar to GLA in ability to activate TLR4, enhances Tfh responses when added to heat-inactivated rabies virus vaccine in a murine model of immunization [68]. Mice immunized with rabies vaccine with MPLA adjuvant had increased co-stimulatory molecule expression on dendritic cells and increased frequencies of Tfh in draining lymph nodes, compared to mice receiving the rabies vaccine without adjuvant. Immunization of mice with rabies vaccine with MPLA led to enhanced frequencies of plasma cells at 2 weeks as well as increased total IgG, and IgG isotype antibody titers. Delivery of antigen in lipid NP may enhance Tfh differentiation with the MPLA adjuvant. In mice immunized with protein antigen in lipid-based synthetic NP vesicles and adjuvanted with MPLA there was significantly enhanced Tfh frequencies induced compared to mice receiving soluble protein antigen with MPLA alone [69]. Further, mice immunized with a *Plasmodium vivax* circumsporozoite antigen, VMP001, formulated as a NP (VMP001-NP) adjuvanted with MPLA demonstrated germinal center formation near depots of VMP001-NPs in draining lymph nodes and had significantly higher IgG responses compared to VMP001-NPs adjuvanted with alum at 400 days after immunization [69].

The MF59 adjuvant is another adjuvant with evidence of enhancing Tfh responses during vaccination. MF59 is an oil-in-water emulsion containing the oil squalene, combined with surfactants and buffered with citric acid. In contrast to the mechanisms of GLA and MPLA based adjuvants, MF59 is believed to act by enhancing numbers of APC recruited to the site of vaccination, influencing total numbers of activated APC in draining lymph nodes [70]. Additionally, the adjuvant effect of MF59 may be dependent on MyD88 adaptor protein activation through a TLR independent signaling mechanism [71]. In mice immunized with influenza hemagglutinin antigen emulsified in MF59, there were significantly increased numbers of Tfh induced on vaccination compared to vaccination without MF59, and frequencies of Tfh correlated with frequencies of B cells within lymph node germinal centers [72]. Similar increases of lymph node germinal center B cells observed as late as 4 months after immunization were noted in a murine model of *Staphylococcus aureus* protein antigen vaccination when adjuvanted with MF59 [73]. However, in human volunteers immunized with the MF59 adjuvanted trivalent influenza vaccine, there was no significant difference in numbers of Tfh observed compared to trivalent influenza vaccine administered without MF59, while numbers of H1N1 specific Tfh significantly correlated with antibody responses at early and late timepoints after vaccination [74]. Interestingly, SARS-CoV-2 spike protein vaccine adjuvanted with MF59 led to the induction of Tfh in the blood in human volunteers at lower doses of vaccine and required booster immunization [75]. Additional studies are needed to determine the potential benefit of MF59 during vaccination in specifically enhancing Tfh responses in humans.

More recently, it has been demonstrated that combination strategies may enhance Tfh induction during vaccination. The synthetic TLR 7/8 agonist, 3M-052, enhances production of Tfh during protein subunit vaccination in macaques when presented in poly(lactic-co-glycolic acid) (PLGA) NP and co-administered with GLA compared to administration of protein subunit vaccination with alum [76]. Of note, in this study, the highest frequencies of long-lived antibody-secreting plasma cells in the bone marrow were identified in macaques that had received protein subunit vaccination with 3M-052 in PLGA NP with or without GLA, compared to vaccine receipt with GLA alone or with alum alone. Hence the combination of 3M-052 formulated in PLGA NP with GLA enhanced generation of Tfh in protein-based vaccination, and was associated with durable antibody-based immune responses.

These studies show promise that TLR4 agonism improves APC presentation of either inactivated virus, peptide, or recombinant protein antigen vaccines, increases T follicular helper cell generation, and enhances B cell and/or class-switched antibody responses (see Table 2). Additionally, MF59 and new synthetic adjuvants, such as 3M-052 targeting TLR7/8, may also play a role in enhancing Tfh induction in future vaccines. New strategies, such as bacterial enzymatic combinatorial chemistry on lipooligosaccharide (as reviewed in [77]) may yield new and diverse TLR4 agonist molecules that may have capacity for further increased Tfh differentiation. Future evaluation of this group of adjuvants to augment vaccination responses in humans should be considered in settings where antibody-based immunity and B cell memory in particular, are critical components of the protective immune response. In settings of immunosuppression where humoral immune responses are suboptimal, such as in the setting of HIV infection or in elderly patients, adjuvants that enhance Tfh responses may play an important role in augmenting B cell maturation and affinity matured, long-lived antibody responses.

## 4. Th1, Th2, and Th17 Immunity and Adjuvants

Naïve CD4+ T cells, upon T cell receptor stimulation and guided by activation of master transcription factors, differentiate into T cells armed with different general types of effector functions. During T cell priming, APC derived cytokines and cell surface co-stimulatory or inhibitory molecules influence this process of T cell differentiation [78]. Hence, the interaction of vaccine or pathogen with APC may critically impact the direction of T-helper (Th) cell subset differentiation. Th1, Th2, and Th17 represent three major CD4+ Th subsets that may be preferentially induced by vaccination and further impacted by adjuvants. The relative importance of these respective Th subsets in host defense diverges significantly depending on the infection. Selective use of adjuvants to enhance optimal human immune defense pathways is a major goal for next generation vaccines.

### 4.1. Th1 Cell Induction and Adjuvants

Th1 cells are generally defined by activation of transcriptional factors T-bet and STAT5, and are characterized by high quantities of IFN-γ production upon T cell receptor (TCR) stimulation. Th1 immunity is associated with enhanced macrophage effector function against pathogens, as well as production of antibody isotypes with ability to enhance Fc-receptor mediated phagocytosis such as IgG1 in humans (the murine homolog of IgG2a). An adjuvant that has shown high ability to drive Th1 responses in vaccination is CpG. In a murine model of vaccination with whole cell lysates of *Leishmania* promastigotes, if the vaccine was administered with CpG there was 10-fold enhanced recruitment of CD4+ T cells producing IFN-γ to the skin upon intradermal *Leishmania* challenge compared to without CpG adjuvant [79], and in a macaque model of this infection the addition of CpG as an adjuvant to a heat-killed *Leishmania* vaccine enhanced protection from disease [80]. In an intranasal model of immunization of influenza in mice, addition of CpG significantly enhanced recruitment of CD4+ T cells in the lung producing IFN-γ, TNF-α, or multifunctional CD4+ T cells producing both cytokines [81]. When serving as an adjuvant to the Hepatitis B protein subunit vaccine in clinical use, CpG is associated with rapid development of high titer antibody responses in humans [82] and is associated with increased levels of seroprotective antibody titers and antibody durability in the setting of HIV infection [83]. The ability of this adjuvant to improve antibody responses in the setting of HIV suggests promise for enhancing antibody responses in other settings of immunodeficiency.

The Adjuvant System (AS) adjuvants, or defined combination adjuvants containing classical and recently identified immunomodulators are being utilized in current and next generation vaccine design. The AS01 and AS04 adjuvants, both licensed for use as adjuvants in humans, are combination adjuvants that have been demonstrated to enhance Th1 immune responses. The AS01 complex is a liposome-based adjuvant containing monophosphoryl A and the saponin QS21. In studies of the *Mycobacterium tuberculosis* modified protein antigen vaccine M72, when adjuvanted with AS01 and administered to volunteers, the vaccine was found to yield high levels of M72 specific CD4+ T cell multifunctional responses with expression of either TNF-α, IFN-γ, or IL-2, and high IFN-γ responses in serum after booster vaccination, compared to vaccine response when using other adjuvants [84]. A malaria vaccine candidate, circumsporozoite-based protein antigen RTS,S when adjuvanted with alum-based adjuvants failed to provide immunologic protection in a malaria controlled human challenge model [85], but when adjuvanted with AS01 provided ~50% vaccine efficacy under controlled human malaria challenge [86]. On analysis of the immune responses elicited by RTS,S adjuvanted with AS01, protected vaccinees had higher levels of antigen specific CD4+ T cells as well as higher levels of IFN-γ producing CD4+ T cells as observed two weeks after booster immunization and at later timepoints. From mechanistic studies, the increased production of Th1 type immune responses mediated by AS01 is suspected to be related to the production of early, innate IFN-γ responses by APCs such as macrophages [87]. Increased APC IFN-γ responses promote a Th1 environment locally, with increased IL-12 and IL-18 expression by APCs influencing downstream programming of T cells in the draining lymph node during APC interaction [87]. Hence the earliest interactions with antigen presenting cells with the adjuvant- vaccine complex may influence the Th-type adaptive immune response observed with this vaccine (see Table 3).

The AS04 complex consists of MPLA adsorbed onto aluminum hydroxide is also associated with enhanced Th1 responses, and is currently in trials in humans for human papillomavirus (HPV) and hepatitis B vaccination [88]. Human dendritic cells stimulated with the HPV vaccine Cervarix, which contains AS04, induced TNF-α and IL-6 production and increased dendritic cell co-stimulatory molecule expression, with the effect found to be specific to the MPLA component [89]. In a murine model of influenza vaccination, virus like particles expressing influenza M2 ectodomain protein (M2eVLP), when adjuvanted with AS04 had superior protective benefit upon lethal influenza challenge after vaccination relative to M2eVLP adjuvanted with alum or MPLA alone or in the absence of any adjuvant [90]. It was noted that M2eVLP vaccination with AS04 also led to higher production of CD4+T cells in the lungs expressing IFN-γ and pore forming protein granzyme B on challenge relative to other groups. These data suggest that the Th1 response induced by the vaccine and recalled with challenge may relate to the mechanism of enhanced immune protection. Additionally, this study demonstrates that features of the combination AS04 are distinct from the separate components as adjuvants. Collectively, these studies underscore the value of AS01 and AS04, and similar adjuvants, in enhancing Th1 responses.

The mRNA COVID-19 vaccines BNT162b2 and mRNA-1273 are associated with a pronounced Th1 response compared to Th2 response in humans. Induction of CD4+ T cells producing either IFN-γ or TNF-α with mRNA-1273 was increased when a higher dose of vaccine was used compared to a lower dose, and notably the magnitude of Th1 response was preserved in adults over the age of 71 compared to adults ages 56–70 [91]. The Th2 response, as assessed by Th production of IL-4 or IL-13 was notably lower and unaffected by vaccine dose or age group. The BNT162b2 vaccine demonstrated a similar polarity towards driving Th1 responses in humans, with minimal IL-4 producing Th responses elicited in subjects at any vaccine dose tested [41]. Given the challenge of inducing reliable T cell responses during vaccination in older adults, these results suggest that the mRNA-lipid NP vaccines may be promising in eliciting strong Th1 responses generally, including in older adults.

### 4.2. Th2 Cell Induction and Adjuvants

Differentiation of Th2 cells is associated with activation of the master transcriptional factor GATA-3, and with Th production of cytokine IL-4, as well as IL-5, IL-9, IL-13, and IL-25. Th2 immunity with an antibody-predominant response is believed to be essential in human defense against parasitic infections, such as in resistance to gastrointestinal worm infections [92,93,94]. There is less known regarding potential Th2 driving adjuvants, compared to Th1 driving adjuvants, perhaps given the success of alum in driving Th2 responses while suppressing Th1 responses (see Table 4). Despite the duration of use of alum as an adjuvant in humans, the mechanism of alum as an adjuvant is unclear. alum’s adjuvant effect is hypothesized to be related to initiating an antigen depot with prolonged recruitment of immune cells, including eosinophils, to the injection site; pattern recognition and activation of the cytosolic NLRP3 inflammasome; and elicitation of endogenous danger signals such as uric acid and IL-33 [95,96]. In a vaccine against Schistosomiasis, protein antigen glutathione *S*-transferase (28GST) derived from *S. mansoni* administered with aluminum hydroxide significantly reduced baboon adult worm quantities and was also associated with reduced infection driven inflammation in the liver [97]. In adult volunteers vaccinated with recombinant *S. hematobium* 28GST adjuvanted with aluminum hydroxide, high levels of 28GST specific IgG1 production were elicited, and PBMCs collected from volunteers after booster dosing and stimulated with 28GST in vitro demonstrated high production of IL-5 and IL-13 consistent with a Th2 dominant response [98]. Similarly, the vaccine for human hookworm, *Necator Americanus* antigen Glutatione-S-Transferase 1 (*Na*-GST-1) was assessed for immunogenicity in humans comparing adjuvants aluminum hydroxide (Alhydrogel) vs. Alhydrogel with glucopyranosyl lipid adjuvant. IgG1 and IgG3 isotypes were predominantly elicited, and there was no increase in antibody titers or rate of response with addition of glycopyranosyl lipid adjuvant over the three dose vaccine series [99]. Other adjuvants, such as cholera toxin, MF59, or polysaccharide based adjuvants, such as glucohexaose analogue beta-glu6, may hold promise in Th2 skewing of the Th response, though several recent studies highlight these groups of adjuvants as having broad capacity to enhance immune responses beyond a Th2 predominant pattern [100,101,102,103,104].

### 4.3. Th17 Cell Induction by Adjuvants

Th17 responses play a critical role in host defense against a variety of bacterial and fungal infections at mucosal sites, as evidenced through disease models and studies in mice and humans with Th17 deficiency [105,106,107,108,109,110]. Beyond host defense mechanisms, Th17 responses are implicated in mechanisms of autoimmune disease [111]. Master transcriptional factor RORγT is essential for Th17 development and function, and Th17 are characterized by production of cytokine IL-17 (also known as IL-17A), and potentially the antimicrobial peptide associated cytokine IL-22 [112,113]. Differentiation of Th17 occurs in the setting of increased quantities of IL-23, IL-6, and IL-1β within the cytokine milieu, suggesting that this environment could potentially be recapitulated by adjuvants to enhance Th17 responses. However, given the relatively recent identification of this subset less is known regarding potential adjuvants driving Th17 differentiation. The cationic liposome adjuvant CAF01 has recently been investigated for its potential to drive Th17 responses. In a murine model of vaccination with tuberculosis fusion protein antigen H28, when adjuvanted with CAF01, it was noted that splenocytes on restimulation with antigen 13 weeks after immunization produced high levels of IL-17A as well as IFN-γ [114]. Furthermore, the IL-17A responses were essentially absent when the H28 was instead adjuvanted with either Alhydrogel, Quil A, or Montanide. The Th17 elicited by the vaccine adjuvanted with CAF01 were found to be stable as a cell population for nearly 2 years, and was capable of activation and recruitment to the lung on *M. tuberculosis* challenge [114]. This study is suggestive of the potential of CAF01 as an adjuvant in driving Th17 memory responses alongside Th1 responses against protein antigens. In mucosal vaccination against tuberculosis in mice with ESAT6 peptide antigen, addition of the *E. coli* type II heat labile enterotoxin as an adjuvant led to the induction of high levels of Th17 cells [115]. In this model, vaccine efficacy was lost in the absence of IL-17 signaling and Th17 cells induced by vaccination were important for the generation of inducible bronchus associated lymphoid tissue (iBALT) in a CXCL13 dependent manner [115]. iBALT induction was hypothesized to play a role in retention of antigen specific T cells within the lungs for rapid effector function upon *M. tuberculosis* exposure. Of note, IL-17 signaling is not required for primary immunity against *M. tuberculosis*, but was shown to have a critical role in mucosal vaccine based immune protection against *M. tuberculosis* elicited with *E. coli* heat labile enterotoxin.

Cholera toxin (CT) has also been shown to enhance Th17 responses in a murine model of anthrax vaccination. When mice were intranasally vaccinated with irradiated *Bacillus anthracis* spores, only if CT was included as an adjuvant with the immunization was there protection against lethal challenge, and blockade of IL-17A reduced survival [116]. On evaluation of the T cells in mice vaccinated with *B. anthracis* spores adjuvanted with CT, there was significantly enhanced generation of CD4+ T cells producing IL-17A compared to IFN-γ or IL-5.

More recently, studies with primary human peripheral blood mononuclear cells (PBMC) have shown that cholera toxin, non-toxic multiple mutated CT (mmCT), and double mutant *E. coli* derived heat labile toxin (dmLT) may be a potential adjuvant in enhancing Th17 differentiation in humans. When each of these modified proteins were introduced into PBMC culture, and T cells activated with Staphylococcal enterotoxin B, significantly enhanced levels of IL-17A were observed [117]. Th17 were specifically identified in the co-culture system through intracellular cytokine staining of CD4+ T cells, and blockade of the IL-1 receptor in PBMC cultures limited IL-17A production, suggesting the importance of IL-1 signaling in Th17 differentiation. Monocytes were identified as upregulating IL-1β production on stimulation with either CT, mmCT, or dmLT, suggesting a potential cellular target for these adjuvants [117].

In a macaque model of intranasal vaccination with Pneumococcal surface protein A (PspA) complexed with cationic cholesteryl group-bearing pullulan nanogel (cCHP nanogel), there was significantly enhanced levels of CD4+ T cells producing IL-17 in the blood, as well as enhanced CD4+T cell production of IL-4 and increased serum antigen-specific IgG1, compared to macaques receiving PspA alone [118]. Mice intranasally vaccinated with PspA presented in cCHP nanogel similarly generate CD4+ T cells that produce IL-17 as well as IL-4 responses and were able to resist a lethal challenge with *Streptococcus pneumoniae* compared to intranasal vaccination with PspA alone [119,120]. Collectively, this data suggests that mucosal vaccination with cCHP enhances Th17, as well as Th2 responses.

More recent work has suggested that activation of the lectin receptor dectin-1 may enhance Th17 differentiation by APCs. Human dendritic cells treated with influenza hemagglutinin antigen bound to an agonist anti-dectin-1 monoclonal antibody induced differentiation of naïve CD4+ T cells towards hemagglutinin antigen specific Th17 cells [121]. Separate studies have shown that dectin-1 agonists zymosan and curdlan polarize human dendritic cells towards induction of Th17 immune responses [122,123]. Additional work is needed to substantiate the role of dectin-1 receptor agonists in eliciting Th17 responses in the setting of vaccination in humans.

Insights into potential benefit of CAF01 liposomes, specific bacterial heat labile enterotoxins, and cCHP nanogels in driving Th17 differentiation suggest future paths for adjuvant design for extracellular mucosal infections (see Table 5). Given the prominence of Th17 in host defense pathways associated with a variety of bacterial pneumonias, mucosal viral infections, such as HIV, as well as fungal infections, such as invasive candidiasis, Th17 driving adjuvants may hold particular benefit for vaccine strategies in these settings.

## 5. Future Directions

The utilization of different adjuvants to drive desired pathways in T cell differentiation is a critical goal for developing effective and long-lasting immunity to infection. Our understandings of the immune pathways activated by different adjuvants, as well as of critical immune pathways in host defense against different infections are informing future directions in adjuvant science and vaccine design. With our continually evolving knowledge of mechanisms of APC pattern recognition and APC activation and signaling, advances in adjuvant design will follow. Progress will also be dependent on implementation of more translational studies of vaccines, with greater emphasis on assessment of immunologic responses to adjuvants, as well as mechanisms of adjuvants, in non-human primates and humans. Scalability and absence of toxicities are also key features in next-generation adjuvants driving T cell differentiation. Additionally, structural manipulation and optimization of adjuvants, such as with the use of bacterial enzymatic combinatorial chemistry on lipooligosaccharide, may hold promise for future adjuvant design. Strategies involving combination adjuvants as well as adjuvants with more specific molecular targets may also help to impact T cell differentiation. The response to an infectious disease in real-time, as illustrated with the COVID-19 pandemic, brought new classes of vaccine and adjuvant into broad use in humans safely and rapidly. Studies of properties of T cell responses from the large populations that have received recently developed adenoviral, protein, and mRNA lipid NP COVID-19 vaccines may pave new directions for adjuvants and delivery platforms for other infectious diseases. In this promising time in vaccinology, advancing the development of adjuvants driving T cell responses in humans may help to address our greatest challenges in infectious disease.

## Figures and Tables

**Table 1:** Adjuvants enhancing CD8+ T cell differentiation in human/non-human primate studies.

Adjuvant Enhancing CD8+ T Cell Differentiation in Human/Non-Human Primate Studies	Potential Mechanism(s)	Pathogen(s)/Antigens for Which the Adjuvant Has Been Tested	Select References
Saponins (e.g., QS-21, ISCOMATRIX *)	Enhances dendritic cell uptake of antigen through receptor-independent, cholesterol-dependent mechanism	Melanoma and prostate cancer protein antigens, bovine diarrhea virus, Ebola virus, SARS-CoV-2	[18,20,21,22,23,25,30]
	Accumulates in and destabilizes, lysosomes, increasing production of cellular activation inflammatory cytokines IL-6 and TNF-α		
Liposomes * mRNA/lipid nanoparticles *	Lysosome destabilization, enhanced cytosolic antigen	Ovalbumin, SARS-CoV-2	[35,36,41,42]
	Induction of CD8+ T cell proliferation		
	Enhance APC processing of vaccine antigen and influence APC maturation		
Live attenuated viruses	Increased antigen load	Yellow fever virus, smallpox virus	[44,45,46,47]
	Broad pattern recognition receptor activation		
	Antigen processing through MHC I		
Viral vectors *	Elicitation of antigen-specific CD8+ T cells, trafficking of antigen into pathways for MHC I expression	Ebola virus, SARS-CoV-2	[48,49,50,51,52,53]
Unmethylated CpG	Stimulation of innate immune system through TLR9 and stimulation of antigen-specific CD8+ T cells	SIV, melanoma antigen A	[55,56]

* An antigen delivery platform with adjuvant functions.

**Table 2:** Adjuvants enhancing Tfh cell subset differentiation in human/non-human primate studies.

Adjuvant Enhancing Tfh Cell Subset Differentiation in Human/Non-Human Primate Studies	Potential Mechanism(s)	Pathogen(s)/Antigens for Which the Adjuvant Has been Tested	Select References
GLA-SE	Activation of TLR4 and enhancement of antigen presentation	Malaria peptide antigens	[65]
3M-052 formulated with PLGA nanoparticles +GLA	Activation of TLR7 and TLR8; with TLR4 activation and nanoparticle-based presentation to APCs	HIV-1 envelope protein	[76]

**Table 3:** Adjuvants enhancing Th1 cell subset differentiation in human/non-human primate studies.

Adjuvant Enhancing Th1 Cell Subset Differentiation in Human/Non-Human Primate Studies	Potential Mechanism(s)	Pathogen(s)/Antigens for Which the Adjuvant Has Been Tested	Select References
Unmethylated CpG	Activation of TLR9	*Leishmania* influenza, hepatitis B	[79,80,81,83]
	Enhanced recruitment of CD4+ T cells		
Adjuvant system AS01 and AS04	Induction of antigen-specific CD4+ T cells	*Mycobacterium tuberculosis*, malaria, human papillomavirus, influenza	[84,86,87,89,90]
	Production of early, innate IFN-γ responses by APCs		
mRNA/lipid nanoparticles *	High level intracellular antigen expression, promoting MHC II antigen presentation	SARS-CoV-2	[41,91]

* An antigen delivery platform with adjuvant functions.

**Table 4:** Adjuvants enhancing Th2 cell subset differentiation in human/non-human primate studies.

Adjuvant Enhancing Th2 Cell Subset Differentiation in Human/Non-Human Primate Studies.	Potential Mechanism(s)	Pathogen(s)/Antigens for Which the Adjuvant Has Been Tested	Select References
Alum	Antigen deposition with prolonged recruitment of immune cells (i.e., eosinophils) to injection site	*Schistomsoma mansoni*, *S. hematobium*, *Necator Americanus*	[93,95,97,98,99]
	Pattern recognition and activation of the cytosolic inflammasome		
	Elicitation of endogenous danger signals (i.e., uric acid, IL-33)		

**Table 5:** Adjuvants enhancing Th17 cell subset differentiation in human/non-human primate studies.

Adjuvant Enhancing Th17 Cell Subset Differentiation in Human/Non-Human Primate Studies	Potential Mechanism(s)	Pathogen(s)/Antigens for Which the Adjuvant Has Been Tested	Select References
Heat labile bacterial toxins and derivatives (e.g., dmLT)	Monocyte upregulation of IL-1β production	*Mycobacterium tuberculosis, Staphylococcus aureus, Bacillus anthracis*	[115,117]
	Induction of iBALT through Th17 and elicitation of CXCR13		
cCHP nanogel *	Particulate mucosal adherent immunization, enhancing local APC activation	*Streptococcus pneumoniae*	[118,119,120]

* An antigen delivery platform with adjuvant functions.

## Data Availability

No new data were created or analyzed in this study. Data sharing is not applicable to this article.
